# Postoperative Outcomes Analysis After Pancreatic Duct Occlusion: A Safe Option to Treat the Pancreatic Stump After Pancreaticoduodenectomy in Low-Volume Centers

**DOI:** 10.3389/fsurg.2021.804675

**Published:** 2021-12-21

**Authors:** Antonio Giuliani, Pasquale Avella, Anna Lucia Segreto, Maria Lucia Izzo, Antonio Buondonno, Mariagrazia Coluzzi, Micaela Cappuccio, Maria Chiara Brunese, Roberto Vaschetti, Andrea Scacchi, Germano Guerra, Bruno Amato, Fulvio Calise, Aldo Rocca

**Affiliations:** ^1^Unit of General and Emergency Surgery, AOR “San Carlo”, Potenza, Italy; ^2^Unit of Hepatobiliary Surgery and Liver Transplant Centre, “Cardarelli” Hospital, Naples, Italy; ^3^Department of Medicine and Health Sciences “V. Tiberio”, University of Molise, Campobasso, Italy; ^4^Department of General Surgery “SS. Antonio e Biagio e Cesare Arrigo” Hospital, Alessandria, Italy; ^5^Department of Clinical Medicine and Surgery, University of Naples Federico II, Naples, Italy; ^6^HPB Surgery Unit, Pineta Grande Hospital, Campania, Italy

**Keywords:** pancreatic surgery, pancreatic cancer, low-volume center, pancreatic stump, duct occlusion, COVID-19 pandemic, POPF

## Abstract

**Background:** Surgical resection is the only possible choice of treatment in several pancreatic disorders that included periampullar neoplasms. The development of a postoperative pancreatic fistula (POPF) is the main complication. Despite three different surgical strategies that have been proposed–pancreatojejunostomy (PJ), pancreatogastrostomy (PG), and pancreatic duct occlusion (DO)–none of them has been clearly validated to be superior. The aim of this study was to analyse the postoperative outcomes after DO.

**Methods:** We retrospectively reviewed 56 consecutive patients who underwent Whipple's procedure from January 2007 to December 2014 in a tertiary Hepatobiliary Surgery and Liver Transplant Unit. After pancreatic resection in open surgery, we performed DO of the Wirsung duct with Cyanoacrylate glue independently from the stump characteristics. The mean follow-up was 24.5 months.

**Results:** In total, 29 (60.4%) were men and 19 were (39.6%) women with a mean age of 62.79 (SD ± 10.02) years. Surgical indications were in 95% of cases malignant diseases. The incidence of POPF after DO was 31 (64.5%): 10 (20.8%) patients had a Grade A fistula, 18 (37.5%) Grade B fistula, and 3 (6.2%) Grade C fistula. No statistical differences were demonstrated in the development of POPF according to pancreatic duct diameter groups (*p* = 0.2145). Nevertheless, the POPF rate was significantly higher in the soft pancreatic group (*p* = 0.0164). The mean operative time was 358.12 min (SD ± 77.03, range: 221–480 min). Hospital stay was significantly longer in patients who developed POPF (*p* < 0.001). According to the Clavien-Dindo (CD) classification, seven of 48 (14.58%) patients were classified as CD III–IV. At the last follow-up, 27 of the 31 (87%) patients were alive.

**Conclusions:** Duct occlusion could be proposed as a safe alternative to pancreatic anastomosis especially in low-/medium-volume centers in selected cases at higher risk of clinically relevant POPF.

## Introduction

Surgical resection is the only possible choice of treatment in several pancreatic disorders, such as malignancies, adenomas, traumas, and severe acute and/or chronic pancreatitis (1). Radical resection is the single most important factor in determining outcomes in patients with pancreatic adenocarcinoma (1–3). Although the surgical context has radically changed in the last 20 years with the advent of new technologies and surgical approaches improving the short-term outcomes in several abdominal surgical fields (4–8), however, the morbidity rate following pancreaticoduodenectomy (PD) remains high, ranging from 30 to 50%, with a mortality rate of 3–5% (9–12). Morbidity in pancreatic surgery is mainly related to the development of a postoperative pancreatic fistula (POPF) (13). According to the International Study Group on Pancreatic Fistula (ISGPF), it is possible to grade POPF based on clinical variables (14). “A grade” fistulas, as called a “biochemical leak” (BL) in update classification, do not need any treatment (currently it is not considered a true pancreatic fistula) and imply no clinical impact. “B grade” fistulas can be managed with medications and only prolong the length of hospital stay in association with a clinically relevant condition. “C grade” fistulas need operative treatment and might be life threatening (12). In high-volume centers for pancreatic surgery, the overall POPF incidence is around 20% (12, 14, 15). Intra-abdominal abscesses, delayed gastric emptying, postpancreatectomy hemorrhage, and sepsis represent additional sources of morbidity. In most cases, however, they occur in association or as a consequence of POPF (16, 17). Advanced age (>75 years), pancreas texture, pancreatic duct diameter, comorbidities, previous endoscopic retrograde cholangiopancreatography (ERCP), duct obstruction, and surgical technique are known risk factors for postoperative morbidity (12, 14, 15, 18–21). The incidence of postoperative complications has a significant impact on the length of hospital stay, costs, quality of life, and chance to start chemotherapy (22, 23). Several different surgical and pharmacological approaches have been proposed to avoid POPF, which might be different depending on the experience and preferences at each center (13, 24). Three main different surgical strategies have been proposed to deal with the pancreatic stump following PD—pancreatojejunostomy (PJ), pancreatogastrostomy (PG) and pancreatic duct occlusion (DO)—but none of them has been clearly demonstrated to be superior to the others (25). Despite such detailed reporting of morbidity and mortality following PD, it is still not clear whether is surgeon's experience or hospital volume to rescue patients when a complication occurs (25). If PJ is the procedure of choice in medium-/high-volume centers, DO could be proposed as a safer alternative in medium-/low-volume centers, to reduce the risk of major postoperative complications (26). We decided to review our previous experience in the light of the recent Covid pandemic where, in our country, it has been forced in many regions to displace treatment of oncological patients in low-volume hospitals with limited experience (27, 28). The encouraging results of DO in terms of overall survival, POPF, and “brittle diabetes” are here presented.

## Materials and Methods

### Study Design

We retrospectively reviewed 56 consecutive patients who underwent Whipple's procedure from January 2007 to December 2014 in a tertiary Hepatobiliary Surgery and Liver Transplant Unit with a low volume of pancreatic resections.

All data were obtained from a prospectively maintained database and analyzed retrospectively. All patients signed a proper informed consent for the scientific anonymous use of clinical data. The study was conducted according to the guidelines of the Declaration of Helsinki and approved by the Institutional Review Board of the University of Molise (protocol number 10/21, approved date: 12 May 2021).

The follow-up program was performed by clinical exam, CEA, CA19.9 levels, and CT scan every 3 of 6 months after surgery according to Italian guidelines (29).

**Figure 1:**
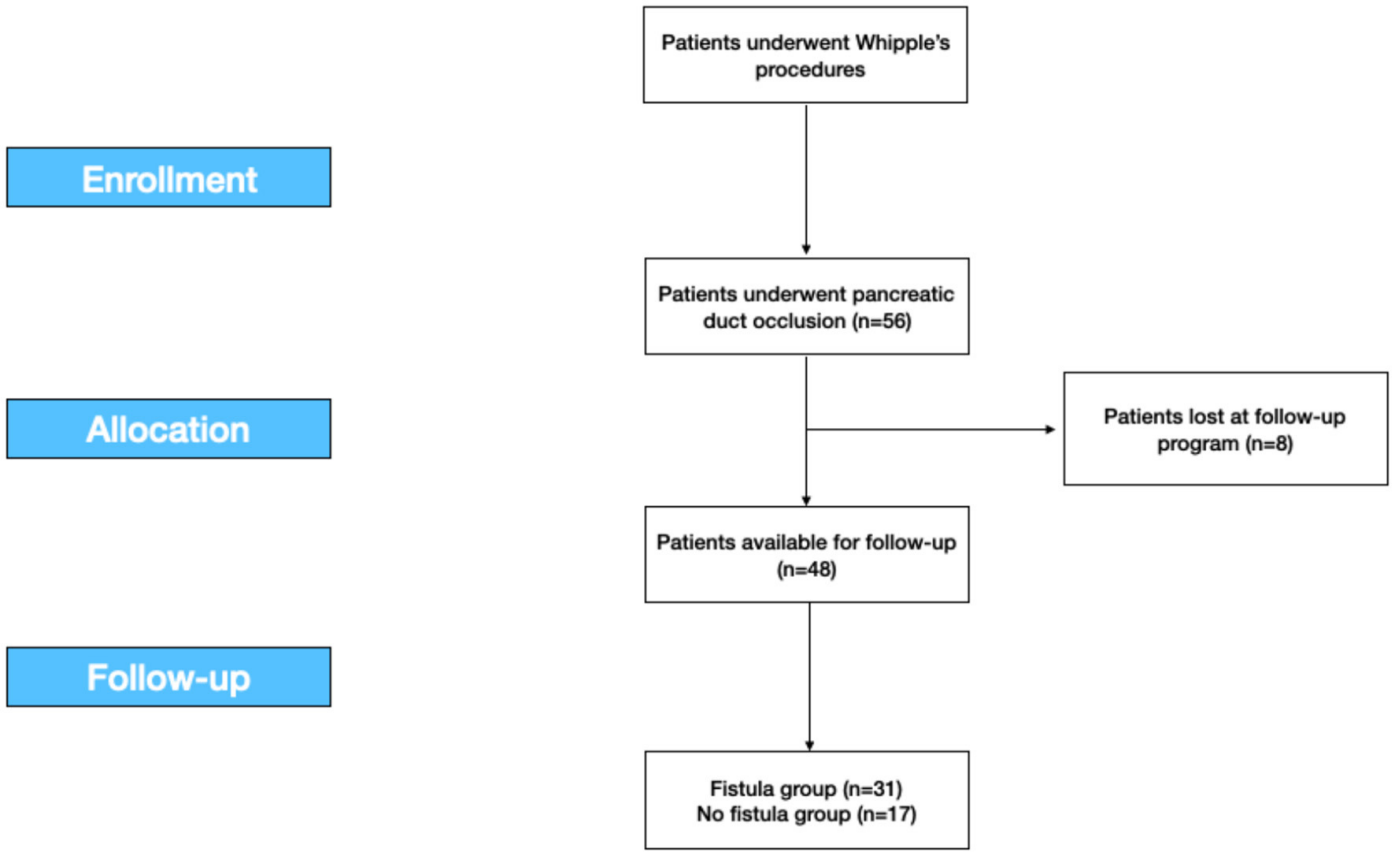
The study flow-chart according to the STROBE statements. STROBE, the Strengthening the Reporting of Observational Studies in Epidemiology.

Eight patients were lost at follow-up, so the analysis on morbidity was conducted on the 48 patients available with a mean follow-up of 25.4 months (Figure 1).

In all cases, DO was performed with Cyanoacrylate glue injection.

We recorded data about medical history, body mass index (BMI), American Society of Anaesthesiologists' (ASA) score, preoperative CA19.9, survival, mean operative time, incidence of POPF, the incidence of sepsis, the incidence of postoperative hemorrhage, re-laparotomy rate, hospital stay, incidence of preoperative and postoperative diabetes, 30-day and 90-day postoperative mortality, oncological recurrence, and pancreatic exocrine function.

The pancreatic exocrine function was evaluated by personal or telephonic interviews assessing any substitutive pancreatic enzyme therapy (yes/no) related to steatorrhea/diarrhea since surgery.

This retrospective study was developed according to the Strengthening the Reporting of Observational Studies in Epidemiology (STROBE) statement for cohort studies (Figure 1) (30).

### Preoperative Workup

Our preoperative workup consisted of total body CT and/or MRI scan for oncological staging and for the exact determination of tumor size and resectability. If total bilirubin was higher than 20 mg/dl, biliary drainage was placed via ERCP in patients whose surgery was not scheduled within 2 wk. A cephalosporin + metronidazole was used as infection prophylactic treatment. No patient was allergic to this regimen.

### Surgical Technique

We performed a Whipple procedure with an open approach. Gastrectomy was performed using GIA 90 without pylorus preservation.

**Figure 2:**
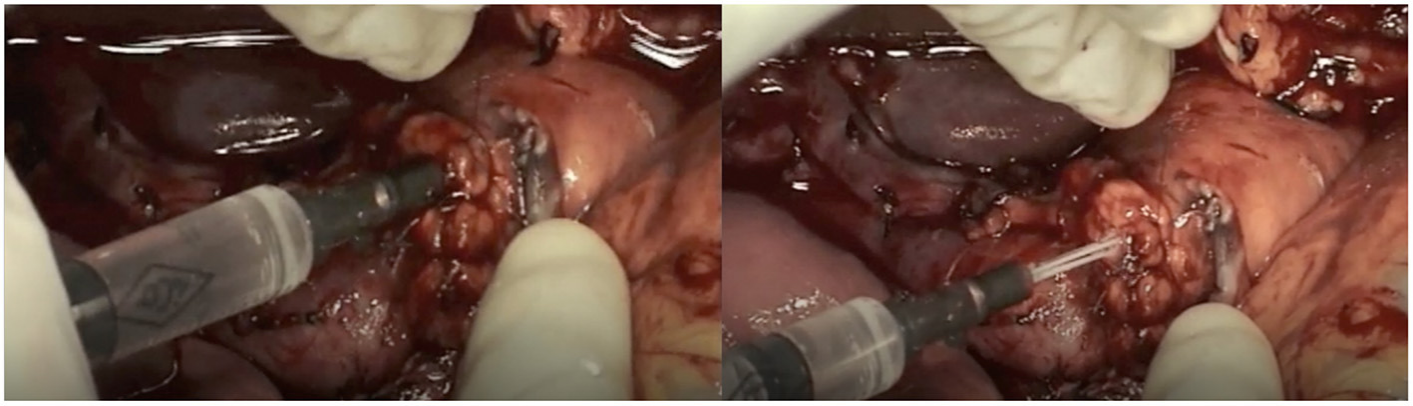
Cyanoacrylate glue injection in Wirsung duct to obtain pancreatic duct occlusion.

After pancreatic resection, we performed DO of the Wirsung duct with Cyanoacrylate glue independently from the stump characteristics. In detail, the pancreatic stump was closed with 3/0 polypropylene stitches during glue polymerization while the catheter inserted in the main pancreatic duct for glue injection was simultaneously removed to obtain a complete duct closure (Figure 2). No patients underwent vascular resection. We finally performed biliary reconstruction with a Roux-en-Y anastomosis. We always performed a mechanical gastro-jejunal anastomosis.

Two abdominal drainages were placed (one close to the pancreatic remnant and one in the pelvis).

### Postoperative Care

All patients stayed at least 1 day in the intensive care unit (range: 1–3 days) and then returned to the ward. Amylase and lipase were routinely monitored in serum starting from postoperative day 3. POPF was defined according to the 2016 update of the International Study Group (ISGPS) (14, 25).

A cephalosporin + metronidazole regimen was adopted when needed. No patient was allergic to this antibiotic regimen and/or presented resistant bacteria. Octreotide 0.1 ml was administered subcutaneously three times a day. In the absence of POPF, patients were allowed oral intake on postoperative day 5.

Complications were graded according to Clavien-Dindo (CD) classification (31).

### Statistical Analysis

Descriptive statistics were collected and reported as a whole number (percentage) and mean or median (range). Chi-square test and Fisher exact test including or not Yates' continuity correction, two-by-two cross tables, Student's *t*-test, and ANOVA test were used to compare categorical data and to analyse normally distributed quantitative data.

Differences were statistically significant when *p*-values were <0.05. Statistical analysis was carried out using IBM SPSS Statistics for Macintosh, Version 27.0.

## Results

For 8 years, from January 2007 to December 2014, we retrospectively collected data of 56 patients who underwent Whipple's procedure for benign and malignant diseases in a Tertiary Hepatobiliary Surgery and Liver Transplant Unit with a low volume of pancreatic resections. Eight patients (8) were excluded upon they were lost at the follow-up program. Total 48 patients were included (Figure 1).

**Figure 3:**
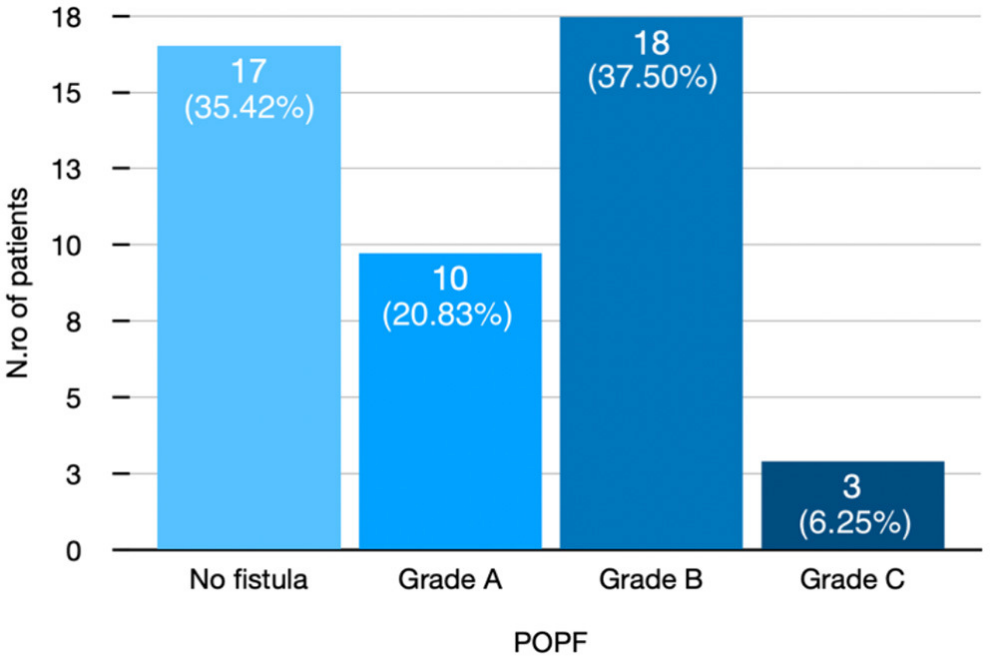
Distribution of patients after pancreatic duct occlusion according to POPF grade. POPF, postoperative pancreatic fistula.

In total, 29 (60.4%) were men and 19 were (39.6%) women with a mean age of 62.79 (SD ± 10.02) years. Thirty-one (64.58%) developed POPF. Figure 3 shows POPF grade in detail.

Body mass index, ASA score, and other baseline characteristics of patients according to the development of pancreatic fistula are shown in Table 1.

**Table 1:** Baseline characteristics of patients who underwent pancreatic duct occlusion.

	**Duct occlusion, =48**	**Fistula, ***n*** = 31**	**No fistula, ***n*** = 17**	***p*** **-value**
**Age (yrs)**				
Mean (± SD)	62.79 (± 10.02)	62.87 (± 8.23)	62.65 (± 12.96)	*0.9429*
Median	66.00	66.00	66.00	
Range	(34–78)	(44–78)	(34–78)	
**Gender,** ***n*** **(%)**				
Male	29 (60.4)	22 (70.97)	7 (41.18)	*0.0651*
Female	19 (39.6)	9 (29.03)	10 (58.82)	
**BMI**				
Mean (± SD)	25.27 (± 1.64)	25 (± 1.54)	25.51 (± 1.71)	*0.2968*
Median	25	25	25	
Range	(21–28)	23–28	21–28	
**ASA,** ***n*** **(%)**				
I	1 (2.1)	1 (3.24)	0 (0)	*1.0000*
II	16 (33.3)	12 (38.71)	4 (23.53)	*0.5316*
III	19 (39.6)	12 (38.71)	7 (41.18)	*1.0000*
IV	12 (25.0)	6 (19.34)	6 (35.29)	*0.3002*
**Previous procedures,** ***n*** **(%)**				
ERCP	16 (33.3)	14 (45.16)	2 (11.76)	*0.486*
PTC stent	2 (4.2)	1 (3.22)	1 (5.88)	*1.0000*
Colecistectomy	1 (2.1)	0 (0)	1 (5.88)	*0.3673*
**Comorbidities,** ***n*** **(%)**				
Arterial hypertension Diabetes mellitus Atrial fibrillation HCV positive COPD Liver transplantation Cerebral ischemia	16 (33.3) 10 (20.8) 6 (12.5) 3 (6.3) 3 (6.3) 1 (2.1) 1 (2.1)	10 (32.26) 5 (16.13) 4 (12.90) 3 (9.68) 2 (6.45) 1 (3.22) 1 (3.22)	6 (35.29) 5 (29.41) 2 (6.45) 0 (0) 1 (5.88) 0 (0) 0 (0)	*1.0000* *0.2947* *1.0000* *0.5430* *1.0000* *1.0000* *1.0000*
**Pre-operative Ca19.9, U/ml**				
Mean, (± SD) Median Range	285.14 (± 660.83) 80.45 (1–2734.10)	117.79 (±85.29) 80.45 (22.4–2431)	787.2 (±1307) 206.85 (1–2734.10)	*0.0062*

*BMI, Body Mass Index; ERCP, Endoscopic Retrograde Cholangiopancreatography; PTC, Percutaneous Transhepatic Cholangiography; COPD, Chronic Obstructive Pulmonary Disease; Ca19.9, carbohydrate antigen 19–9 or cancer antigen 19–9 or sialylated Lewis*.

Surgical indications were in 95% of cases malignant diseases. Pathological findings according to POPF are depicted in Table 2.

**Table 2:** Clinico-pathological data of patients who underwent pancreas duct occlusion included in follow-up program.

	**Duct occlusion, ***n*** = 48**	**Fistula, ***n*** = 31**	**No fistula, ***n*** = 17**	***P*** **-value**
**Histological findings,** ***n*** **(%)**				
Pancreatic adenocarcinoma Ampullary adenocarcinoma Bile duct cancer Neuroendocrin carcinoma Mucinous cystadenoma Gallbladder cancer Choronic pancreatitis	24 (50) 10 (20.84) 6 (12.50) 3 (6.25) 3 (6.25) 1 (2.08) 1 (2.08)	14 (45.16) 6 (19.35) 5 (16.14) 2 (6.45) 3 (9.68) 1 (3.22) -	10 (58.83) 4 (23.53) 1 (5.88) 1 (5.88) - - 1 (5.88)	*0.5469* *0.7266* *0.4022* *1.0000* *0.5430* *1.0000* *0.3542*
**Pancreatic texture,** ***n*** **(%)**				
Soft Hard Normal	33 (68.75) 8 (16.67) 7 (14.58)	25 (80.65) 5 (16.13) 1 (3.22)	8 (47.06) 3 (16.65) 6 (35.39)	*0.0164* *1.0000* *0.0055*
**Pancreatic duct diameter**				
Mean, mm Range, mm ≤ 3 mm, *n* (%) > 3mm, *n* (%)	3.98 (± 2.18) 1–10 19 (39.58) 29 (60.42)	4.25 (± 1.88) 3–10	5.00 (± 2.14) 1–8	*0.2145*
**Hematic amylase, UI/l**				
Pre-operative mean (± SD) Post-operative, mean (± SD) 7 days p.o., mean (± SD)	178.41 (± 201.37) 451.31 (± 510.78) 74.10 (± 57.44)	202.75 (±236.89) 557.37 (±567.52) 88.10 (±60.90)	139.47 (±123.41) 246.33 (±298.06) 47.93 (±40.21)	*0.3110* *0.0413* *0.0187*

Biliary drainage was performed before surgery in 16 (33.3%) patients who underwent ERCP, in one patient (4.2%) who underwent PTC. The incidence of pancreatic fistula after biliary drainage is shown in Table 1.

Duct diameter was reported larger than 3 mm in 60% of patients. As depicted in Table 2, no statistical differences were demonstrated in the development of POPF according to pancreatic duct diameter groups (*p* = 0.2145).

The soft pancreatic texture was recorded in 68% of cases. As shown in Table 2, the POPF rate was significantly higher in the soft pancreatic group (*p* = 0.0164).

The mean operative time was 358.12 min (SD ± 77.03, range: 221–480 min). Six (12.5%) patients needed intraoperative blood cells transfusions (Table 3).

**Table 3:** Perioperative data.

**Operative time, min**	
Mean (± SD) Median Range	358.12 (± 77.03) 360 221–480
**Procedures, ***n*** (%)**	
Glubran	48 (100)
**Blood trasfusion**	
*n* (%) packed red blood cells, mean (range)	6 (12.5) 1.5 (1–4)
**Hospital stay, days, mean (± SD)**	
Fistula group No fistula group *p*-value	38 (± 22), (r.:13–115) 17.37 (± 9), (r.:3–45) < 0.001

Hospital stay was significantly longer in patients who developed POPF (*p* < 0.001) as described in Table 3.

**Figure 4:**
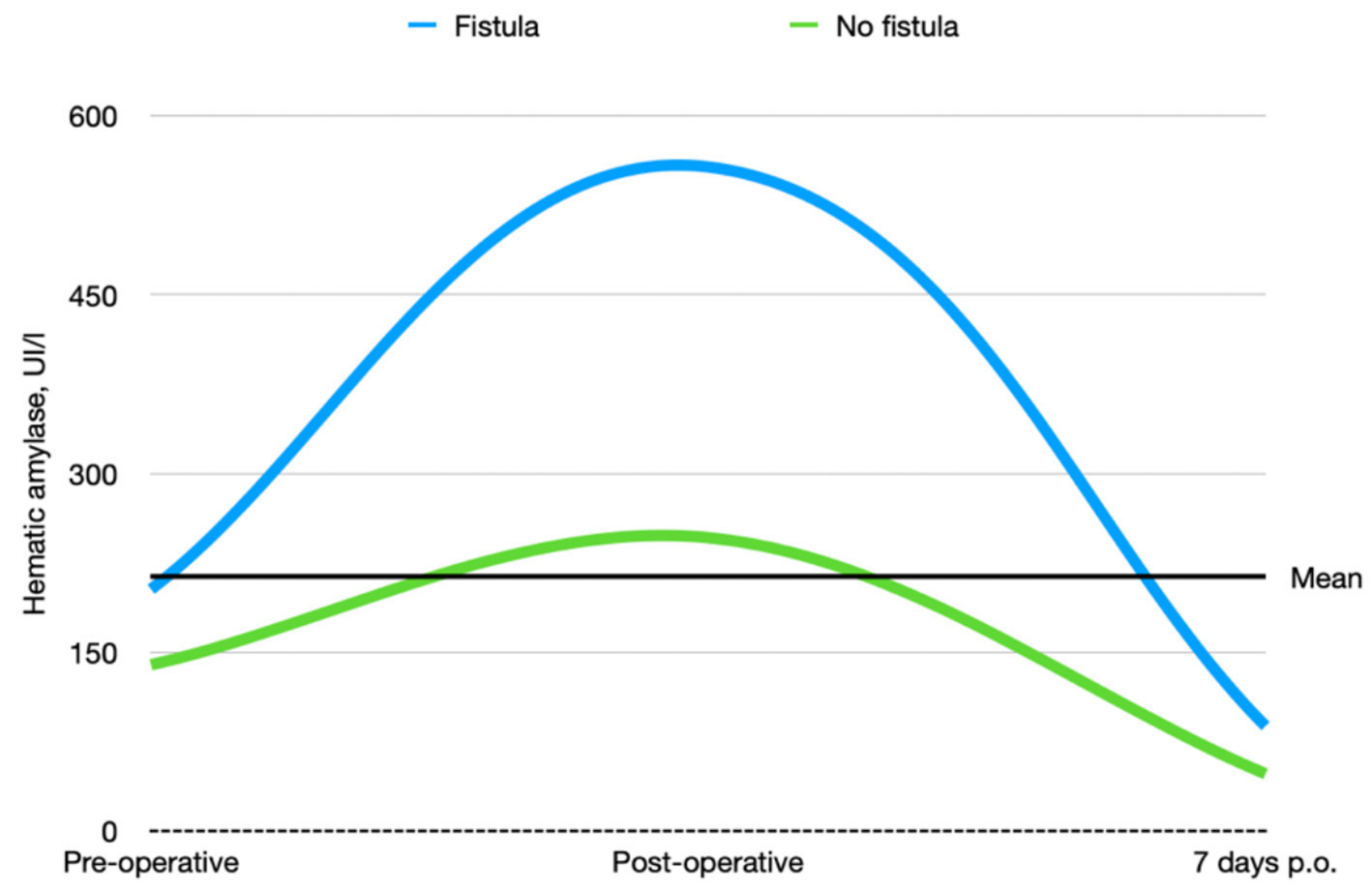
Preoperative, postoperative, and 7-day postoperative hematic amylase trends in patients who underwent pancreatic duct occlusion with and without fistula.

According to the CD classification (31), seven of 48 (14.58%) patients were classified as CD III–IV. Complications, reoperation rate, and whole short-term outcomes that include 30- and 90-day mortality according to pancreatic fistula are extensively described in Tables 4–6 and Figure 4.

**Table 4:** Short-term and long-term outcomes.

	**Duct occlusion, ***n*** = 48**	**Fistula, ***n*** = 31**	**No fistula, ***n*** = 17**	***P*** **-value**
**Clavien-Dindo classification,** ***n*** **(%)**				
I–II III–IV	41 (85.42) 7 (14.58)	27(87.10) 4 (12.90)	14 (82.35) 3 (17.65)	*0.6862*
30-days mortality, *n* (%)	3 (6.45)	2 (6.45)	1 (5.88)	*1.0000*
90-days mortality, *n* (%)	2 (4.16)	2 (6.45)	0	*1.0000*
**Short-term outcomes,** ***n*** **(%)**				
Sepsis Post-operative bleeding Intraddominal collection Pleura effusion Dehiscence* Hemoperitoneum Intestinal obstruction Stroke DIC	11 (22.92) 10 (20.83) 14 (29.17) 2 (4.17) 2 (4.17) 4 (8.33) 2 (4.17) 1 (2.08) 2 (4.17)	9 (29.03) 9 (29.03) 14(45.16) 1 (3.22) 1 (3.22) 2 (6.45) 2 (6.45) 1 (3.22) 2 (6.45)	2 (11.76) 1 (5.88) 0 1 (5.88) 1 (5.88) 2 (11.76) 0 0 0	*0.2840* *0.0744* * < 0.001* *1.0000* *1.0000* *0.2300* *0.5328* *1.0000* *0.5328*
**Long-term outcomes,** ***n*** **(%)**				
Brittle diabetes Octreotide therapy	8 (16.67) 44 (91.67)	3 (9.68) 31 (100)	5 (29.41) 13 (76.47)	*0.1115* *0.0122*
**Reoperative rate,** ***n*** **(%)**				
Total Hemostatis Total pancreasectomy GI fistula Re-anastomosis HJ Explorative laparotomy	10(20.83) 4 (8.33) 2 (4.17) 1 (2.08) 1 (2.08) 1 (2.08)	7 (22.58) 2 (6.45) 2 (6.45) 1 (3.22) 1 (3.22) 1 (3.22)	2 (11.76) 2 (11.76) . . . .	*0.6073* *0.5328* *1.0000* *1.0000* *1.0000*
Recurrence, *n* (%)	7 (14.58)	6 (19.35)	1 (5.88)	*0.3956*
**Follow-up, months**				
Mean Range	24.5 (3–100)	23.5 (3–100)	17.7 (3–21)	
Overall survival (%)	58.3			

DIC, disseminated intravascular coagulation; HJ, Hepatico-Jejunostomy;*Dehiscence: 1 Hepatico-jejunostomy; 1 wound.

**Table 5:** Mortality rate and cause of death.

	**POPF grade**	**Cause of death**
**30-days mortality, n.ro**		
1 1 1	No POPF A grade C grade	Shock-MOFS MOFS Stroke
**90-days mortality, n.ro**		
1 1	A grade B grade	Hemorrhage-MOFS MOFS

*POPF, postoperative pancreatic fistula*.

**Table 6:** Re-operative rate according to POPF grade and follow-up.

	**POPF grade**	**Follow-up**
**Hemostasis, n.ro**		
1 1 1 1	No POPF No POPF A grade A grade	Dead 30 days p.o. Alive 12 months p.o. Dead 7 months p.o. Alive 78 months p.o.
**Total pancreasectomies, n.ro**		
1 1	C grade C grade	Dead 30 months p.o. Alive 100 months p.o.
**GI fistula, n.ro**		
1	C grade	Alive 8 months p.o.
**Re-anastomosis hepatico-jejunal, n.ro**		
1	C grade	Alive 27 months p.o.
**Explorative laparotomy, n.ro**		
1	C grade	Dead 90 days p.o.

*POPF, postoperative pancreatic fistula*.

Eight (16.67%) patients developed brittle diabetes without any statistical relationship to the POPF rate (Table 4).

The mean follow-up was 24.5 months (range: 3–100; Table 4).

The overall survival at the last follow-up was 58.3% (Table 4).

## Discussion

Our case series demonstrate that DO might be considered as a safe option to treat pancreatic stump after PD. Evidence supports a strong correlation between surgical outcomes and hospital volume in pancreatic surgery (32–37). Despite these findings during the Covid pandemic period, it was very difficult to provide sanitary migration to high-volume centers (38–40), so also medium- and low-volume centers, which have enough facilities and skills to provide pancreatic surgery, should perform more interventions to answer to the population needs. Our results gained in a Hepatobiliary referral center with a low-volume rate of pancreatic resections may encourage pancreatic resection allowing a reduction of patient mobility. Pedrazzoli et al. in a large systematic review on PD and pancreatic fistula analyzed 162 articles involving 54,232 patients (41). The review shows 4,813 Grade A (8.9%), 4,830 Grade B (8.9%), and 1,872 Grade C (3.5%) POPFs with a mean overall fistula rate of 21.3%. A huge variability of Grades A and B POPFs varied from <2% to more than 20% with a minimum of 0% and a maximum of 42.5% for Grade A and a minimum of 0.7% and a maximum of 33.3% for Grade B POPF. Grade C POPFs arise from 1% to more than 9% with a maximum of 13.6% (41). Di Carlo et al. showed that the DO procedure was feasible and less time-consuming than PJ, although it could be associated with higher fistula rates. However, POPF could not be clinically relevant probably due to the absence of a pancreatic enzymes activation (42). In our experience, the overall incidence of POPF was 64%. This observation is consistent with the experience of Tersigni et al. who observed a higher rate of POPF after DO (45.4%) compared to end-to-end PJ anastomosis (15.6%) and to end-to-side PJ anastomosis (11.3%), with a similar incidence of Grade C fistula in all the groups (3.1% after end-to-end PJ anastomosis, 2.3% after end-to-side anastomosis and 3.0% after DO) (43). Consistent with other reports, in our patients a soft pancreatic texture was associated with a significantly higher incidence of POPF (overall 80% of POPF with soft pancreas vs. 16% of POPF with fibrotic pancreas). Moreover, when considering only clinically relevant POPF, we had only two POPFs (4.2%) with fibrotic pancreas vs. 15 POPFs (31.4%) with the soft pancreas (*p* < 0.005). Our incidence of reoperation was quite high 9/48, 18.7% (Table 4). It is superimposable to Duffas et al. and Mazzaferro et al. (26, 44). In detail, if we consider patients re-operated due to POPF only in two cases the prognosis was poor. Five re-operated patients had a good prognosis, so we can consider that the stump treatment did not influence the reoperation rate. Four of our patients (8.3%) had a postoperative hemorrhage, and all of them needed to return to the operative room. Interestingly, in only two patients (50%) hemorrhage was a consequence of POPF (all grade A). In the other two cases, the bleeding originated from a small vessel from the portal vein and the gastroepiploic artery. The overall incidence of POPF-related bleeding was 6%, which is in line with other experiences (25). Our length of stay was 38 days in POPF-group, higher than those observed in other experiences (45). More than 90% of patients needed pancreatic enzymes supplementation due to postoperative pancreatic insufficiency. This facet is consistent with other authors (25, 46, 47). However, Tran et al. reported that the need for enzyme supplementation 1 year after surgery was not related to the type of reconstruction (46). In addition, other authors reported that pancreatic exocrine insufficiency might be related to the pancreatic atrophy/fibrosis and preoperative texture than to DO or PJ (25, 46, 47). In our series, 16% of patients developed brittle diabetes, with only 13 patients (27.1%) developing new-onset diabetes. This might confirm that DO has a higher risk of new-onset diabetes, even if only a few patients suffer from uncontrolled diabetes (25, 46, 47). According to Tran et al., the incidence of endocrine insufficiency is significantly higher after DO compared with PJ at 3- and 12-month follow-up after surgery (*p* = 0.001 for both) (46). The overall mortality rate in more than 1,500 PD performed in Italy was reported to be as high as 8.1% (34). Our findings are superimposable to the literature (34), but we would clarify that only two patients who died have developed a clinically relevant fistula. On the other hand, three patients died for cardiovascular causes despite the absence of B or C POPF. We also demonstrated an overall pancreatic surgery-related mortality, which is lower than for low-volume centers (34). It has been suggested that avoiding an anastomosis of the pancreatic duct by means of duct occlusion could minimize anastomosis-related morbidity, especially in low-volume centers (43, 46–48). The aim was to obtain a “*pure”* pancreatic fistula with no activation by bile and/or enteric juice, thereby reducing the risk of life-threatening complications. However, in the experience of a high-volume center, postoperative mortality after PJ seemed to be higher than after DO (43). In a recent prospective randomized control study (26) compared POPF following DO in high-risk patients for pancreatic fistula vs. PJ after PD for low-risk patients for pancreatic fistula, mortality after DO was 5.9% and 2.0% after PJ anastomosis, in our serie 90-day mortality related to significant POPF was (2/48) 4%, so mortality might be considered superimposable with other authors who performed DO (Table 4) (49). He et al. (33) analyzed Randomized Controlled Trials (RCTs) and Observational Clinical Studies (OCSs), which were related to different treatments of pancreatic stump and major outcomes after PD or pylorus-preserving PD for malignant or benign pancreatic tumor, chronic pancreatitis, or extra-pancreatic tumors (periampullary, biliary or duodenal). The objective of the meta-analysis was a comparison between PJ and PG using quantitative data on POPF and overall complications. PD without anastomosis or duodenum-preserving pancreatectomy was excluded. We shall underline meta-analysis by He et al. (33) reported a lower mortality index performing PG and PJ, but these data were published by high volume and referral centers for pancreatic surgery. Nevertheless, Duffas et al. reported in their experience an incidence of death after PG and PJ of 12 and 10%, respectively (44). A summary of these findings is depicted in Tables 7–9.

**Table 7:** Literature summary of pathological findings in pancreatic surgery.

**Author**	**Type**	**N.ro**	**Mean Operative Time, min (range)**	**PA,** **n (%)**	**Amp,** **n (%)**	**BDC,** **n (%)**	**Others, n (%)**	**Texture soft, n (%)**	**DD≤3mm, n (%)**
Giuliani et al.	DO	48	358 (r.:221–480)	24 (50)	10 (20.8)	6 (12.5)	8 (16.6)	33 (68.7)	19 (39.58)
Mazzaferro et al. (26)	DO	51	480 (r.:400–533)	33 (64.7)	32 (65.3)	6 (10.7)	5 (9.8)	NA	NA
	PJ	49	490 (r.:438–540)	32 (65.3)	4 (8.2)	6 (10.7)	7 (14.3)	NA	NA
Yeo (50)	PG	73	444 (r.:432–456)	40 (55)	7 (10)	6 (8)	4 (5)	16 (22)	3,4 (mean)
	PJ	72	432 (r.:420–444)	40 (56)	11 (15)	7 (10)	7 (9.7)	17 (24)	2,9 (mean)
Duffas (44)	PG	81	≥360 54 (67%) <360 27 (33%)	34 (42)	17 (19)	8 (10)	9 (11)	49 (60)	32 (40)
	PJ	68	≥360 44 (65%) <360 24 (35%)	25 (37)	19 (28)	11 (16)	8 (11.7)	41 (60)	49 (60)
Bassi (51)	PG	69	337.2 (r:336-338)	32 (46)	13 (18.8)	1 (1.4)	24 (34.7)	NA	NA
	PJ	82	353.9 (r: 352-354)	28 (34.1)	11 (13.4)	2 (2.4)	43 (52.4)	NA	NA
Fernàndez-Cruz (52)	PG	53	300 (r.:250-350)	26 (49)	12 (22.6)	8 (15)	10 (18.8)	24 (45)	NA
	PJ	55	310 (r.:250-370)	28 (50.9)	10 (18.1)	7 (12.7)	10 (18.1)	25 (55)	NA

*PA, pancreatic adenocarcinoma; Amp, ampullary carcinoma; BDC, bile duct cancer; DD, duct diameter; DO, duct occlusion; PJ, pancreatic-jejunal anastomosis; PG, pancreatic-gastrostomy; NA, not available*.

**Table 8:** Literature summary of complications in pancreatic surgery.

**Author**	**Type**	**N.ro**	**P.O. haemorrhage, ***n*** (%)**	**SI, ***n*** (%)**	**Pneumonia, ***n*** (%)**	**Bleeding, ***n*** (%)**	**BF, ***n*** (%)**	**IA, ***n*** (%)**	**DGE, ***n*** (%)**
Giuliani et al.	DO	48	8 (16.67)	4 (8.3)	3 (6.2)	11 (22.9)	1 (2)	14 (29.17)	NA
Mazzaferro et al. (26)	DO	51	7 (13.7)	5 (9.8)	8 (15.7)	7 (13.7)	4 (7.8)	4 (7.8)	8 (15.7)
	PJ	49	5 (10.2)	2 (4.1)	7 (14.3)	5 (10)	7 (14.3)	2 (4.1)	9 (18.4)
Yeo (50)	PG	73	NA	14 (19)	5 (7)	NA	1 (1)	4 (5)	16 (22)
	PJ	72	NA	11 (15)	2 (3)	NA	3 (4)	2 (3)	16 (22)
Duffas (44)	PG	81	13 (16)	NA	NA	13 (16)	6 (7)	11 (14)	NA
	PJ	68	9 (13)	NA	NA	9 (13)	2 (3)	16 (23)	NA
Bassi (51)	PG	69	3 (4)	NA	NA	3 (4)	0	7 (10)	2 (3)
	PJ	82	6 (7)	NA	NA	6 (7)	7 (8.5)	22 (27)	10 (12)
Fernàndez-Cruz (52)	PG	53	1 (2)	3 (8)	2 (4)	1 (2)	0	2 (4)	2 (4)
	PJ	55	1(2)	2 (4)	4 (7)	1 (2)	1 (2)	8 (14)	8 (14)

*SI, surgical infection; BF, biliary fistula; IA, intra-abdominal abscess; DO, duct occlusion; DGE, Delayed Gastric Emptying; PJ, pancreatic-jejunal anastomosis; PG, pancreatic-gastrostomy; NA, not available*.

**Table 9:** Literature summary of Clavien-Dindo classification, re-operative rate, POPF and mortality rate in pancreatic surgery.

**Author**	**Type**	**N.ro**	**CD I–II, ***n*** (%)**	**CD ≥III, ***n*** (%)**	**Re-operation rate, ***n*** (%)**	**POPF, ***n*** (%)**	**Mortality, ***n*** (%)**
Giuliani et al.	DO	48	41 (85)	7 (14)	10 (20.83)	31 (64.5)	5 (10.4)
Mazzaferro et al. (26)	DO	51	15 (29.4)	36 (70.6)	9 (19)	B, C 6 (11.8)	3 (5.9)
	PJ	49	15 (30.6)	34 (69.4)	8 (16.3)	B, C 8 (16.3)	1 (2)
Yeo (50)	PG	73	NA	NA	NA	9 (12)	NA
	PJ	72	NA	NA	NA	8 (11)	NA
Duffas (44)	PG	81	44 (54.3)	37 (45.7)	15 (19)	13 (16)	10 (12)
	PJ	68	38 (55.9)	30 (44.1)	15 (22)	14 (20.5)	7 (10)
Bassi (51)	PG	69	NA	NA	5 (7)	9 (15.8)	0
	PJ	82	NA	NA	5 (6)	13 (15.8)	1 (1)
Fernàndez-Cruz (52)	PG	53	NA	NA	1 (1.8)	A:1 (1,8) B:2 (3.7)	0
	PJ	55	NA	NA	1 (1.8)	B:10 (18.1)	0

*CD, Clavien-Dindo Classification; NA, not available*.

It is clear that the outcome of complex surgical procedures may not only rely on technical aspects of surgery but is also affected by resource availability (53, 54). However, some technical aspects can be modified and reduce the risk of life-threatening postoperative complications even in low-/medium-volume centers. Pancreaticoduodenectomy can be safely performed in low-volume centers if amenities and processes typical of high-volume centers can be replicated in specialized units (55, 56). Of note, we represent the only referral center for HPB in a huge geographical region of southern Italy, so the availability of postdischarge home management, financial problems, low human resources and patients wish could affect this outcome. In our opinion, in patients with a higher risk for POPF (soft pancreas, dilated pancreatic duct), DO could be a safer option, ideally suitable in low-volume centers. The ideal concept of reserving pancreatic surgery only to highly specialized centers is probably *utopian*. Geographical limitations, elevated costs for the patients and their relatives, political issues, different regional healthcare systems, and the opposition by medical and surgical staff determine the need to perform this surgery even in academic or tertiary referral hospitals with a limited experience in HPB surgery, but with all the amenities required for very complex surgery (57, 58). So, considering criteria published in the literature (32, 34–36), pancreatic surgery should be centralized, this implies unavoidably an increase of interregional mobility and related healthcare costs, especially for patients from the region of southern Italy. During the Covid-19 pandemic, as we know from the survey written by Aldrighetti et al. on HPB surgery in Italy (27), 72.8% of HPB centers showed a reduction of routine elective operations ≥50%, if we combine effects of centralization to the effects of the Covid-19 pandemic we understand how difficult it would be for patients to undergo pancreatic surgery in a quite fast, safe, and effective way (59). In this situation, we decided to analyse our outcomes from a low volume center for pancreatic surgery to overcome the impossibility to send patients to pancreatic surgery referral centers, considering their overload, ensuring to patients a high-quality service at the same time. Our approach led us to guarantee effective treatment and safety procedures during the critical pandemic period. Probably, a surgical alternative such as DO during the phase of PD at higher risk of complications, i.e., the pancreatic anastomosis, could reduce the rates of subsequent morbidity and mortality with similar oncological results.

### Limitations

Our study is a retrospective, single-center analysis, we considered consecutive patients who underwent PD and were registered in a prospectively maintained database. We can consider our center as low volume due to the number of PD per year, but we can be supported by high-volume center facilities, including a) being a referral center for hepatobiliary surgery, liver transplantation, advanced colorectal surgery, b) having a dedicated intensive care unit, and c) having interventional radiology and endoscopy available 24 h.

## Conclusions

In conclusion, DO could be proposed as an alternative option to pancreatic anastomosis especially in low-/medium-volume centers. A comparison of DO with other types of pancreatic duct reconstructions should be advisable to draw definitive conclusions, ideally by means of an adequately designed RCT.

## Data Availability Statement

The raw data supporting the conclusions of this article will be made available by the authors, with undue reservation.

## Ethics Statement

The studies involving human participants were reviewed and approved by Università degli Studi del Molise. The patients/participants provided their written informed consent to participate in this study.

## Author Contributions

AG, FC, and AR: conceptualization. AG and AR: methodology. PA: software. AG, PA, FC, and AR: validation, writing—original draft preparation, and visualization. PA, ALS, MI, AB, MCo, MCa, MB, RV, and AS: formal analysis and investigation, resources, and data curation. PA, FC, and AR: writing—review and editing. GG, BA, and AR: supervision. All authors have read and agreed to the published version of the manuscript.

## Conflict of Interest

The authors declare that the research was conducted in the absence of any commercial or financial relationships that could be construed as a potential conflict of interest.

## Publisher's Note

All claims expressed in this article are solely those of the authors and do not necessarily represent those of their affiliated organizations, or those of the publisher, the editors and the reviewers. Any product that may be evaluated in this article, or claim that may be made by its manufacturer, is not guaranteed or endorsed by the publisher.
